# Fibrinous Concretion of the Heart

**Published:** 1849-04

**Authors:** 


*Fibrinous Concretion of the Heart.*—On the 18th of April, 1848, Mr. Harrinson presented a specimen of diseased heart, taken from a patient, who, having previously enjoyed pretty good health, began to complain of debility, lassitude, and obscure chest symptoms. Suddenly he was attacked with extreme difficulty of breathing; the skin greatly congested; blue from head to foot; surface cold; pulse scarcely to be felt; heart beating rapidly, and rather forcibly; orthopnœa; cough; expectoration of viscid mucus mixed with blood;—*not* hæmoptysis. Crepitation was heard at various points, and percussion was dull. He sunk gradually.

*Autopsy* sixteen hours after death. General surface still dingy; about a pint of straw-colored fluid in each plural cavity; the lungs gorged with black fluid blood; the heart filled the pericardial bag; right auricle enormously distended with blood, the ventricle comparatively empty; left cavities distended with black blood and fibrinous deposit. The auricle was hypertrophied, and almost filled with a mass of vegetation, nearly closing the auriculo-ventricular opening, only a small orifice remaining posteriorly. The vegetations were made up of large granules, intimately adherent with each other, and connected with the auricular surface of the valves, forming a solid vascular nucleus. The ventricular surface of the valves were perfectly free. Liver large and gorged.

On examining the fungus, it appeared to be more a fibrinous deposit than any vegetation from the valves, for the serous membrane of the valves elsewhere, did not exhibit any trace of inflammation, and it was probably deposited there on some unknown nucleus.—*Prov. Med. Journal, England.*

